# Emergent Dynamical Properties of the BCM Learning Rule

**DOI:** 10.1186/s13408-017-0044-6

**Published:** 2017-02-20

**Authors:** Lawrence C. Udeigwe, Paul W. Munro, G. Bard Ermentrout

**Affiliations:** 1 Department of Mathematics, Manhattan College, 4513 Manhattan College Parkway Riverdale, New York, 10471 USA; 2 School of Information Science, University of Pittsburgh, 135 North Bellefield Avenue, Pittsburgh, PA 15260 USA; 3 Department of Mathematics, University of Pittsburgh, 301 Thackeray Hall, Pittsburgh, PA 15260 USA

**Keywords:** BCM, Learning rule, Oscillation, Chaos

## Abstract

The Bienenstock–Cooper–Munro (BCM) learning rule provides a simple setup for synaptic modification that combines a Hebbian product rule with a homeostatic mechanism that keeps the weights bounded. The homeostatic part of the learning rule depends on the time average of the post-synaptic activity and provides a sliding threshold that distinguishes between increasing or decreasing weights. There are, thus, two essential time scales in the BCM rule: a homeostatic time scale, and a synaptic modification time scale. When the dynamics of the stimulus is rapid enough, it is possible to reduce the BCM rule to a simple averaged set of differential equations. In previous analyses of this model, the time scale of the sliding threshold is usually faster than that of the synaptic modification. In this paper, we study the dynamical properties of these averaged equations when the homeostatic time scale is close to the synaptic modification time scale. We show that instabilities arise leading to oscillations and in some cases chaos and other complex dynamics. We consider three cases: one neuron with two weights and two stimuli, one neuron with two weights and three stimuli, and finally a weakly interacting network of neurons.

## Introduction

For several decades now, the topic of synaptic plasticity has remained relevant. A pioneering theory on this topic is the Hebbian theory of synaptic modification [1, 2], in which Donald Hebb proposed that when neuron A repeatedly participates in firing neuron B, the strength of the action of A onto B increases. This implies that changes in synaptic strengths in a neural network is a function of the pre- and post-synaptic neural activities. A few decades later, Nass and Cooper [3] developed a Hebbian synaptic modification theory for the synapses of the visual cortex, which was later extended to a threshold dependent setup by Cooper et al. [4]. In this setup, the sign of a weight modification is based on whether the post-synaptic response is below or above a static threshold. A response above the threshold is meant to strengthen the active synapse, and a response below the threshold should lead to a weakening of the active synapse.

One of the widely used models of synaptic plasticity is the Bienenstock–Cooper–Munro (BCM) learning rule with which Bienenstock et al. [5]—by incorporating a dynamic threshold that is a function of the average post-synaptic activity over time—captured the development of stimulus selectivity in the primary visual cortex of higher vertebrates. In corroborating the BCM theory, it has been shown that a BCM network develops orientation selectivity and ocular dominance in natural scene environments [6, 7]. Although the BCM rule was developed to model selectivity of visual cortical neurons, it has been successfully applied to other types of neurons. For instance, it has been used to explain experience-dependent plasticity in the mature somatosensory cortex [8]. Furthermore the BCM rule has been reformulated and adapted to suit various interaction environments of neural networks, including laterally interacting neurons [9, 10] and stimuli generalizing neurons [11]. The BCM rule has also been in the center of the discussion as regards the relationship between rate-based plasticity and spike-time dependent plasticity (STDP); it has been shown that the applicability of the BCM formulation is not limited to rate-based neurons but under certain conditions extends to STDP-based neurons [12–14].

Based on the BCM learning rule, a few data mining applications of neuronal selectivity have emerged. It has been shown that a BCM neural network can perform projection pursuit [7, 15, 16], i.e. it can find projections in which a data set departs from statistical normality. This is an important finding that highlights the feature detecting property of a BCM neural model. As a result, the BCM neural network has been successfully applied to some specific pattern recognition tasks. For example Bachman et al. [17] incorporated the BCM learning rule in their algorithm for classifying radar data. Intrator et al. developed an algorithm for recognizing 3D objects from 2D view by combining existing statistical feature extraction models with the BCM model [18, 19]. There has been a preliminary simulation on how the BCM learning rule has the potential to identify alpha numeric letters [20].

Mathematically speaking, the BCM learning rule is a system of differential equations involving the synaptic weights, the stimulus coming into the neuron, the activity response of the neuron to the stimulus, and the threshold for the activity. Unlike its predecessors, which use static thresholds to modulate neuronal activity, the BCM learning rule allows the threshold to be dynamic. This dynamic threshold provides stability to the learning rule, and from a biological perspective, provides homeostasis to the system. Treating the BCM learning rule as a dynamical system, this paper explores the stability properties and shows that the dynamic nature of the threshold guarantees stability only in a certain regime of homeostatic time scale. This paper also explores the stability properties as a function of the relationship between homeostasis time scale and the weight time scale. Indeed, there is no biological reason why the homeostatic time scale should be dramatically shorter than the synaptic modification time scale [21], so in this paper, we relax those restrictions. In Sect. 3, we illustrate a stochastic simulation in the simplest case of a single neuron with two weights and two different competing stimuli. We derive the averaged mean field equations and show that there are changes in the stability as the homeostatic time constant changes. In Sect. 4, we continue the study of a single neuron, but now assume that there are more inputs than weights. Here, we find rich dynamics including multiple period-doubling cascades and chaotic dynamics. Finally, in Sect. 5, we study small linearly coupled networks and prove stability results while uncovering more rich dynamics.

## Methods

**Fig. 1:**
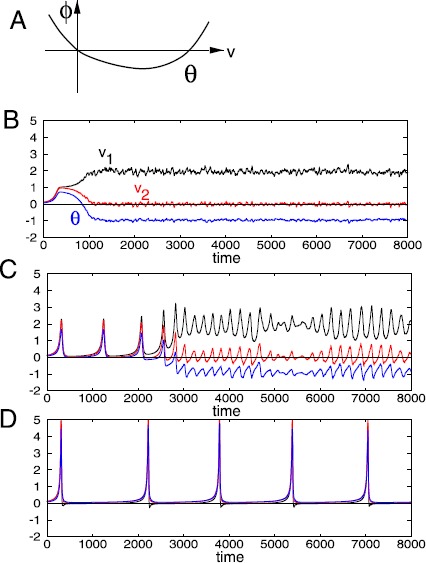
(**A**) A nonlinear function *ϕ* of the post-synaptic neuronal activity, *v*, and a threshold *θ*, of the activity. (**B**) When $\tau_{\theta}/ \tau_{w} =0.25$, response converges to a steady state and neuron selects stimulus $\mathbf{x}^{(1)}$. (Here, the stimuli are $\mathbf{x}^{(1)}=(\cos\alpha,\sin\alpha)$ and $\mathbf{x}^{(2)}=(\sin\alpha,\cos\alpha)$ with $\alpha=0.3926$, the stimuli switch randomly at a rate 5, and $\tau_{w}=25$.) (**C**) When $\tau_{\theta}/ \tau_{w} =1.7$, responses oscillate but the neuron still selects stimulus $\mathbf{x}^{(1)}$. (**D**) When $\tau_{\theta}/ \tau_{w} =2.5$, neuron is no longer selective

The underlying BCM theory expresses the changes in synaptic weights as a product of the input stimulus pattern vector, **x**, and a function, *ϕ*. Here, *ϕ* is a nonlinear function of the post-synaptic neuronal activity, *v*, and a dynamic threshold, *θ*, of the activity (see Fig. 1A).

If at any time, the neuron receives a stimulus **x** from a stimulus set, say $\{ \mathbf{x}^{(1)}, \mathbf{x}^{(2)},\ldots ,\mathbf{x}^{(n)}\}$, the weight vectors, **w**, evolve according to the BCM rule as

1 $$ \begin{aligned} \frac{d\mathbf{w}}{dt} &= \phi(v; \theta)\mathbf{x}, \\ \theta&= E^{p}[v], \end{aligned} $$

*θ* is sometimes referred to as the “sliding threshold” because, as can be seen from Eq. (1), it changes with time, and this change depends on the output *v*, the sum of the weighted input to the neuron, $v=\mathbf{w}\cdot\mathbf{x}$. *ϕ* has the following property: for low values of the post-synaptic activity $(v<\theta)$, *ϕ* is negative; for $v>\theta$, *ϕ* is positive. In the results presented by Bienenstock et al. [5], $\phi(v) = v(v-\theta)$ is used, $E[v]$ is a running temporal average of *v* and the learning rule is stable for $p>1$. Later formulations of the learning rule (for instance by [7]) have shown that a spatial average can be used in lieu of a temporal average, and that with $p=2$, $E[v^{p}]$ is an excellent approximation of $E^{p}[v]$. We can also replace the moving temporal average of *v* with first order low-pass filter. Thus a differential form of the learning rule is

2 $$ \begin{aligned} \tau_{w}\frac{d\mathbf{w}}{dt} &= v \mathbf{x}(v-\theta), \\ \tau_{\theta}\frac{d\theta}{dt} &= \bigl(v^{2} - \theta\bigr), \end{aligned} $$

where $\tau_{w}$ and $\tau_{\theta}$ are time-scale factors, which in simulated environments, can be used to adjust how fast the system is changing with respect to time. We point out that this is the version of the model that is found in Dayan and Abbott [22]. We point out that the vector input, **x** is changing rapidly compared to *θ* and **w**, so that Eq. (2) is actually a stochastic equation. The stimuli, **x** are generally taken from a finite set of patterns, $\mathbf{x}^{(k)}$ and are randomly selected and presented to the model.

## Results I: One Neuron, Two Weights, Two Stimuli

For a single linear neuron that receives a stimulus pattern $\mathbf {x}=(x_{1}, \ldots,x_{n} )$ with synaptic weights $\mathbf{w}=(w_{1},\ldots ,w_{n})$, the neuronal response is $v=\mathbf{w} \cdot\mathbf{x}$. The results we present in this section are specific to when $n=2$ and when there are two patterns. In this case, the neuronal response is $v = {w_{1}}{x_{1}} + {w_{2}}{x_{2}}$. In the next section, we explore a more general setting.

### Stochastic Experiment

A good starting point in studying the dynamical properties of the BCM neuron is to explore the steady states of *v* for different time-scale factors of *θ*. This is equivalent to varying the ratio $\tau _{\theta}/ \tau_{w}$ in Eq. (2). We start with a BCM neuron that receives a stimulus input **x** stochastically from a set $\{ \mathbf{x}^{(1)}, \mathbf{x}^{(2)}\}$ with equal probabilities, that is, $Pr[\mathbf{x}(t)=\mathbf{x}^{(1)} ]=Pr[\mathbf{x}(t)=\mathbf {x}^{(2)} ]= \frac{1}{2}$. We create a simple hybrid stochastic system where the value of **x** switches between the pair $\{ \mathbf{x}^{(1)}, \mathbf{x}^{(2)}\}$ at a rate *λ* as a two state Markov process. At steady state, the neuron is said to be selective if it yields a high response to one stimulus and a low (≈0) response to the other.

Figures 1B–D plot the neuronal response *v* as a function of time. In each case, the initial conditions of $w_{1}$, $w_{2}$ and *θ* lie in the interval $(0,0.3)$. The stimuli are $\mathbf{x}^{(1)}=(\cos\alpha,\sin\alpha)$ and $\mathbf{x}^{(2)}=(\sin\alpha,\cos\alpha)$ where $\alpha=0.3926$. $v_{1}= \mathbf{w} \cdot x^{(1)}$ is the response of the neuron to the stimulus $\mathbf {x}^{(1)}$ and $v_{2}= \mathbf{w}\cdot x^{(2)}$ is the response of the neuron to the stimulus $\mathbf{x}^{(2)}$. In each simulation, the presentation of stimulus is a Markov process with rate $\lambda= 5$ presentations per second. When $\tau_{\theta}/ \tau_{w}= 0.25$, Fig. 1B shows a stable selective steady state of the neuron. At this state, $v_{1} \approx2$ while $v_{2}\approx0$, implying that the neuron selects $\mathbf{x}^{(1)}$. This scenario is equivalent to one of the selective steady states demonstrated by Bienenstock et al. [5].

When the threshold, ***θ*** changes slower than the weights, *w*, the dynamics of the BCM neuron take on a different kind of behavior. In Fig. 1C, $\tau_{\theta}/ \tau_{w}=1.7$. As can be seen, there is a difference between this figure and Fig. 1B. Here, the steady state of the system loses stability and a noisy oscillation appears to emerge. The neuron is still selective since there is a large enough empty intersection between these ranges of oscillation.

Setting the time-scale factor of *θ* to be a little more than twice that of **w** reveals a different kind of oscillation from the one seen in Fig. 1C. In Fig. 1D where $\tau _{\theta}/ \tau_{w}=2.5$, the oscillation has very sharp maxima and flat minima and can be described as an alternating combination of spikes and rest states. As can be seen, the neuron is not selective.

### Mean Field Model

The dynamics of the BCM neuron (Eq. (2)) is stochastic in nature, since at each time step, the neuron randomly receives one out of a set of stimuli. One way to gain more insight into the nature of these dynamics is to study a mean field deterministic approximation of the learning rule. If the rate of change of the stimuli is rapid compared to that of the weights and threshold, then we can average over the fast time scale to get a mean field or averaged model and then study this through the usual methods of dynamical systems. Consider a BCM neuron that receives a stimulus input **x**, stochastically from the set $\{ \mathbf{x}^{(1)}=(x_{11}, x_{12}), \mathbf{x}^{(2)} =(x_{21}, x_{22})\}$ such that $Pr[\mathbf{x}(t)=\mathbf{x}^{(1)} ]= \rho$ and $Pr[\mathbf{x}(t)=\mathbf{x}^{(2)} ]=1-\rho$. A mean field equation for the synaptic weights is

$$\dot{w_{i}}=\rho x_{1i} v_{1}(v_{1}- \theta) + (1-\rho) x_{2i} v_{2}(v_{2}-\theta),\quad i \in\{1,2\}. $$

Now let the responses to the two stimuli be $v_{1} = \mathbf{w} \cdot \mathbf{x}^{(1)}$ and $v_{2} = \mathbf{w} \cdot\mathbf{x}^{(2)}$. With this, changes in the responses can be written as

3 $$ \begin{aligned} \dot{v}_{1} &= x_{11} \dot{w}_{1} + x_{12} \dot{w}_{2} ,\\ \dot{v}_{2} &= x_{21} \dot{w}_{1} + x_{22} \dot{w}_{2} . \end{aligned} $$

So a mean field equation in terms of the responses is

4 $$ \begin{aligned} \tau_{w}\dot{v}_{1} &= \bigl[ \rho\mathbf{x}^{(1)}\cdot\mathbf{x}^{(1)} v_{1}(v_{1} -\theta) + (1-\rho) \mathbf{x}^{(1)}\cdot\mathbf{x}^{(2)} v_{2}(v_{2} -\theta)\bigr], \\ \tau_{w}\dot{v}_{2} &= \bigl[\rho\mathbf{x}^{(1)} \cdot\mathbf{x}^{(2)} v_{1}(v_{1} -\theta) + (1-\rho) \mathbf{x}^{(2)}\cdot\mathbf{x}^{(2)} v_{2}(v_{2} -\theta)\bigr], \\ \tau_{\theta}\dot{\theta} &= \bigl[ \rho{v_{1}}^{2} + (1-\rho) {v_{2}}^{2} -\theta\bigr]. \end{aligned} $$

This equation is our starting point for the analysis of the effects of changing the time-scale factor of *θ*, $\tau_{\theta}$. Thus all that matters with regard to the time scales is the ratio, $\tau=\tau _{\theta}/\tau_{w}$. We note that we could also write down the averaged equations in terms of the weights, but the form of the equations is much more cumbersome.

We now look for equilibria and the stability of these fixed points. We note that if the two stimuli are not collinear and $\rho\in(0,1)$, then $\dot{v}_{1,2}=0$ if and only if $v_{j}(v_{j}-\theta)=0$. Using the fact that at equilibrium, $\theta=\rho{v_{1}}^{2} +( 1-\rho) {v_{2}}^{2}$, we find

5 $$ \begin{aligned} v_{1}\bigl(v_{1} - \bigl( \rho{v_{1}}^{2} +( 1-\rho) {v_{2}}^{2} \bigr)\bigr) &= 0, \\ v_{2}\bigl(v_{2} - \bigl(\rho{v_{1}}^{2} +( 1-\rho) {v_{2}}^{2} \bigr)\bigr) &= 0, \end{aligned} $$

which gives the fixed points

6 $$ (v_{1}, v_{2}, \theta) = \biggl\{ (0, 0, 0), \biggl(\frac {1}{\rho}, 0, \frac{1}{\rho} \biggr), \biggl( 0, \frac{1}{1-\rho }, \frac {1}{1-\rho} \biggr), (1, 1, 1) \biggr\} . $$

The fixed points $(\frac{1}{\rho}, 0, \frac{1}{\rho} )$ and $( 0, \frac{1}{1-\rho}, \frac{1}{1-\rho} )$ are stable (as we will see) for small enough *τ* and selective, while $(0,0,0)$ and $(1,1,1)$ are neither stable nor selective. Bienenstock et al. [5] discussed the stability of these fixed points as they pertain to the original formulation. Castellani et al. [9] and Intrator and Cooper [7] gave a similar treatment to the objective formulation. In Sect. 3.4, it will be shown that the stability of $(\frac {1}{\rho}, 0, \frac{1}{\rho} )$ and $( 0, \frac {1}{1-\rho}, \frac{1}{1-\rho} )$ depends on the angle between the stimuli, the amplitude of the stimuli, *ρ*, and the ratio of $\tau_{\theta}$ to $\tau_{w}$.

### Oscillatory Properties: Simulations

As seen in the preceding section, the fixed points to the mean field BCM equation are invariant (with regards to stimuli and synaptic weights) and depend only on the probabilities with which the stimuli are presented. The stability of the selective fixed points, however, depends on the time-scale parameters, the angular relationship between the stimuli, and the amplitudes of the stimuli. To get a preliminary understanding of this property of the system, consider the following simulations of Eq. (4); each with different stimulus set characteristics. We remark that because Eq. (4) depends only on the inner product of stimuli, equal rotation of both has no effect on the equations. What matters is the magnitude, angle between them, and frequency.

*Simulation A: orthogonal, equal magnitudes, equal probabilities*

**Fig. 2:**
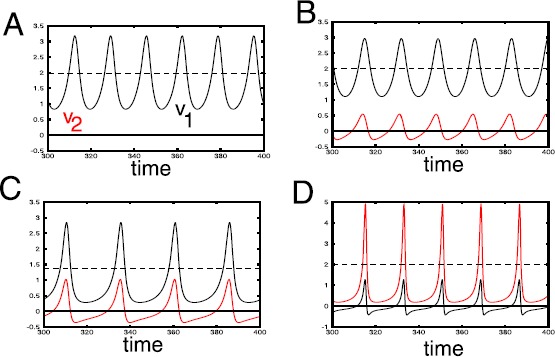
Four simulations of Eq. (4) with initial data $(v_{1},v_{2},\theta)=(0.1,0,0)$ shown for the last 100 time units. $\tau _{w}=2$, $\mathbf{x}^{(1)}=(1,0)$. Equilibria are $v_{2}=0$ and $v_{1}=1/\rho$, shown as the *dashed line*. (**A**) $\rho=0.5$, $\mathbf{x}^{(2)}=(0,1)$, $\tau _{\theta}/\tau_{w}=1.1$; (**B**) $\rho=0.5$, $\mathbf{x}^{(2)}=(\cos (1),\sin(1))$, $\tau_{\theta}/\tau_{w}=1.5$; (**C**) $\rho=0.7$, $\mathbf{x}^{(2)}=(\cos (1),\sin (1))$, $\tau_{\theta}/\tau_{w}=1.5$; (**D**) $\rho=0.5$, $\mathbf {x}^{(2)}=1.5(\cos (1),\sin(1))$, $\tau_{\theta}/\tau_{w}=0.8$

Let $\rho=0.5$, $\mathbf{x}^{(1)} = (1,0)$, $\mathbf{x}^{(2)} = (0,1)$. In this case, the two stimuli have equal magnitudes, are perpendicular to each other, and are presented with equal probabilities. Figure 2(A) shows the evolution of $v_{1}$ and $v_{2}$ in the last 100 time-steps of a 400 time step simulation. The dashed line shows the unstable non-zero equilibrium point. For $\tau\equiv\tau_{\theta}/\tau _{w}=1.1$, there is a stable limit cycle oscillation of $v_{1}$. Since the stimuli are orthogonal, $v_{2}(t)=0$ is an invariant set.

*Simulation B: non-orthogonal, equal magnitudes, equal probabilities*

Let $\rho=0.5$, $\mathbf{x}^{(1)} = (1,0)$, $\mathbf{x}^{(2)} = (\cos(1),\sin (1))$, $\tau=1.5$. In this case, the two stimuli have equal magnitudes, are not perpendicular to each and are presented with equal probabilities. Figure 2(B) shows an oscillation, but now $v_{2}$ oscillates as well since the stimuli are not orthogonal.

*Simulation C: non-orthogonal, equal magnitudes, unequal probabilities*

Let $\rho=0.7$, $\mathbf{x}^{(1)} = (1,0)$, $\mathbf{x}^{(2)} = (\cos(1),\sin(1))$. The only difference between this case and simulation B is that the stimuli are now presented with unequal probabilities. For $\tau=1.5$, there is a stable oscillation of both $v_{1},v_{2}$ centered around their unstable equilibrium values.

*Simulation D: orthogonal, unequal magnitude, equal probabilities*

Let $\rho=0.5$, $\mathbf{x}^{(1)} = (1,0)$, $\mathbf{x}^{(2)} = 1.5(\cos(1), \sin(1))$. The only difference between this case and simulation B is that stimulus 2 has a larger magnitude and $\tau=0.8$. We remark that in this case, even when $\tau<1$, the equilibrium point has become unstable.

These four examples demonstrate that there are oscillations of various shapes and frequencies that arise pretty generically no matter what the specifics of the mean field model are; they can occur in symmetric cases (e.g. simulation A) or with more general parameters as in B-D. We also note that to get oscillatory behavior in the BCM rule, we do not even need $\tau_{\theta}> \tau_{w}$ as seen in example D. We will see shortly that the oscillations arise from a Hopf bifurcation as the parameter, *τ* increases beyond a critical value. To find this value, we perform a stability analysis of the equilibria for Eq. (4).

### Stability Analysis

We begin with a very general stability theorem that will allow us to compute stability for an arbitrary pair of vectors and arbitrary probabilities of presentation. Looking at Eq. (4), we see that by rescaling time, we can assume that $\mathbf{x}^{(1)}\cdot \mathbf{x}^{(1)}=1$ without loss of generality. To simplify the calculations, we let $\tau=\tau_{\theta}/\tau_{w}$, $b= \mathbf{x}^{(1)}\cdot \mathbf{x}^{(2)}$, $a=\mathbf{x}^{(2)}\cdot\mathbf{x}^{(2)}$, and $c=\rho/(1-\rho)$. Note that $a>b^{2}$ by the Schwartz inequality and that $c\in(0,\infty)$ with $c=1$ being the case of equal probability.

For completeness, we first dispatch with the two non-selective equilibria, $(1,1,1)$ and $(0,0,0)$. At $(1,1,1)$, it is easy to see that the characteristic polynomial has a constant coefficient that is $\rho(1-\rho)(b^{2}-a)/\tau$, which means that it is negative since $a>b^{2}$. Thus, $(1,1,1)$ is linearly unstable.

Linearization about $(0,0,0)$ yields a matrix that has double zero eigenvalue and a negative eigenvalue, $-1/\tau$. Since the only linear term in Eq. (4) is $-\theta/\tau$, the center manifold is parameterized by $(v_{1},v_{2})$ and first terms in a center manifold calculation for *θ* are $\theta=\rho v_{1}^{2}+(1-\rho)v_{2}^{2}$. This term only contributes cubic terms to the $v_{1},v_{2}$ right-hand sides so that to quadratic order:

$$\begin{aligned} v_{1}' &= \rho v_{1}^{2} + (1-\rho) b v_{2}^{2} , \\ v_{2}' &= \rho b v_{1}^{2} + (1-\rho) a v_{2}^{2}. \end{aligned}$$

Hence,

$$\frac{dv_{1}}{dv_{2}} = \frac{c + b (v_{2}/v_{1})^{2}}{c b + a (v_{2}/v_{1})^{2}}. $$

We claim that there exists a solution to this equation of the form, $v_{2}=Kv_{1}$ for a constant $K>0$. Plugging in this assumption we see that *K* satisfies

$$\frac{1}{K} = \frac{c + b K^{2}}{cb + a K^{2}}\equiv H(K). $$

For $b>0$, there is a unique $K>0$ satisfying this equation. (Note $b>0$ means the vectors form an acute angle with each other. If $b<0$ then $H(K)$ has a singularity and there is still a root to $H(K)=1/K$. If $b=0$, then there is also a unique solution.) Plugging $v_{2}=Kv_{1}$ into the equation for $v_{1}'$ yields

$$v_{1}' = \bigl(\rho+(1-\rho)b K^{2}\bigr) v_{1}^{2}. $$

If $b\ge0$, then clearly $v_{1}(t)$ goes away from the origin, which implies that $(0,0,0)$ is unstable. If $b<0$, the singularity occurs when $K^{2}=-cb/a$ and the root to $H(K)=1/K$ is less than $-cb/a$. This yields

$$v_{1}' > (1-\rho) \bigl(c -cb^{2}/a \bigr)v_{1}^{2} = \rho\bigl(1-b^{2}/a \bigr)v_{1}^{2} $$

and, again, using the fact that $b^{2}< a$, we see that $v_{1}$ leaves the origin. Thus, we have proven that $(0,0,0)$ is unstable.

We now have to look at the stability of the selective equilibria: $(v_{1},v_{2},\theta)=(1/\rho,0,1/\rho)\equiv\mathbf{z}_{1}$ and $(v_{1},v_{2},\theta )=(0,1/(1-\rho),1/(1-\rho))\equiv\mathbf{z}_{2}$, since the latter has different stability properties if the parameter $a>1$. The Jacobian matrix for the right-hand sides of Eq. (4) around $\mathbf {z}_{1}$ is

$$J= \begin{pmatrix} 1 & -bc & -1 \\ b & -ac & -b \\ 2/\tau& 0 & -1/\tau \end{pmatrix}. $$

From this we get the characteristic polynomial:

$$p_{J}^{1}(\lambda) = \lambda^{3} + A_{12} \lambda^{2}+ A_{11} \lambda+ A_{10}, $$

where

$$\begin{aligned} A_{10} &= c \bigl(a-b^{2}\bigr)/\tau, \\ A_{11} &= (1+ac)/\tau+ c \bigl(b^{2}-a\bigr), \\ A_{12} &= 1/\tau+ ac -1. \end{aligned}$$

Equilibria are stable if these three coefficients are positive and from the Routh–Hurwitz criterion, $A_{11}A_{12}-A_{10}:=R_{1}>0$. We note that $A_{10}>0$ since $c>0$ (unless $\rho=0$) and $a>b^{2}$. This means that no branches of fixed points can bifurcate from the equilibrium point; that is there are no zero eigenvalues. For *τ* small $R_{1}\sim (1+ac)/\tau^{2}>0$ and the other coefficients are positive, so the rest state is asymptotically stable. A Hopf bifurcation will occur if $R_{1}=0$ and $A_{10}>0$ and $A_{12}>0$. Setting $R_{1}=0$ yields the quadratic equation:

7 $$\begin{aligned} \tau^{2}R_{1}&\equiv Q_{R}^{1}( \tau) \\ &=c\bigl(a-b^{2}\bigr) (1-ac)\tau^{2} - \bigl(1+2ac-a^{2}c^{2}-2b^{2}c\bigr)\tau+(1+ac)=0. \end{aligned}$$

In the “standard” case (e.g. as in Fig. 2B), we have $a=c=1$ and $Q_{R}^{1}(\tau)=-2(1-b^{2})\tau+2 =0$ or

8 $$ \tau=1/\bigl(1-b^{2}\bigr). $$

A similar calculation can be done for the fixed point $\mathbf{z}_{2}$. In this case, the coefficients of the characteristic polynomial are

$$\begin{aligned} A_{20} &= c \bigl(a-b^{2}\bigr)/\tau, \\ A_{21} &= (a+c)/\tau+c\bigl(b^{2}-a\bigr), \\ A_{22} &= 1/\tau+c-a. \end{aligned}$$

As with the equilibrium $\mathbf{z}_{1}$, there can be no zero eigenvalue and $A_{20}$ is positive except at the extreme cases where $c=0$ or $a=b^{2}$. The Routh–Hurwitz quantity, $R_{2}:=A_{21}A_{22}-A_{20}$ vanishes at roots of

9 $$ \tau^{2} R_{2}\equiv Q_{R}^{2}( \tau)= c\bigl(a-b^{2}\bigr) (a-c)\tau ^{2}+\bigl(2c \bigl(b^{2}-a\bigr)+c^{2}-a^{2}\bigr)\tau+ a+c =0. $$

We note that when $a=c=1$, we recover Eq. (8). For *τ* sufficiently small, $\mathbf{z}_{2}$ is asymptotically stable.

We now use Eqs. (7) and (9) to explore the stability of the two solutions as a function of *τ*. We have already eliminated the possibility of losing stability through a zero eigenvalue since both $A_{10},A_{20}$ are positive. Thus, the only way to lose stability is through a Hopf bifurcation which occurs when either of $Q_{R}^{1,2}(\tau )$ vanishes. We can use the quadratic formula to solve for *τ* for each of these two cases, but one has to be careful since the coefficient of $\tau^{2}$ vanishes when $c=a$ or $c=1/a$.

**Fig. 3:**
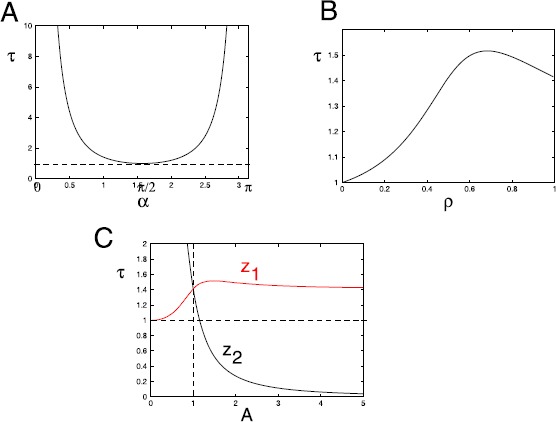
The critical value of $\tau=\tau_{\theta}/\tau_{w}$ for a Hopf bifurcation to equations 4. For $\tau>\tau_{c}$, the selective equilibrium point is unstable. (**A**) Dependence on *α*, the angle between the stimulus vectors when $\rho=0.5$ and the amplitudes of both stimuli are 1. (**B**) Dependence on *ρ* when the amplitudes are 1 and $\alpha=1$. (**C**) Dependence on the amplitude, *A*, of the second stimulus ($a=A^{2}$), $\rho=0.5$, and $\alpha=1$. Note that the stability depends on the equilibria; *red* corresponds to $(2,0,2)$ and *black* to $(0,2,2)$. *Horizontal dashed lines* show $\tau=1$ and the *vertical dashed line* is the equal amplitude case

Figure 3 shows stability curves as different parameters vary. In panel A, we use the standard setup (Fig. 2B) where $\rho=0.5$, the stimuli are unit vectors ($(1,0)$ and $(\cos \alpha ,\sin\alpha)$), and *α* denotes the angle between the vectors. The curve is explicitly obtained from Eq. (8), with $b=\cos \alpha$. For any *τ* above $\tau_{c}$, either of the two selective equilibria is unstable. In Fig. 3B, we show the dependence of $\tau_{c}$ on *ρ*, the frequency of a given stimulus. All values of $\tau_{c}$ are greater than or equal to 1, so that in order to get instability the time-scale factor, $\tau_{\theta}$, of homeostasis must be more than or equal to that of the weights, $\tau_{w}$. In panel C, we show the dependence on the amplitude, *A*, where $\mathbf {x}^{(2)}=A(\cos \alpha,\sin\alpha)$. This figure shows two curves: the red curve give $\tau_{c}$ for the equilibrium, $(v_{1},v_{2},\theta)=(2,0,2)$ while the black curve is for $(v_{1},v_{2},\theta)=(0,2,2)$. The latter equilibrium can lose stability at arbitrarily low values of *τ* if the amplitude is large enough. Indeed, $\tau_{c} \sim1/A^{2}$ as $A\to\infty$.

We summarize the results in this section with the following theorem.

#### Theorem 3.1

*Assume that the two stimuli are not collinear and that* $\rho \in(0,1)$. *Then there are exactly four equilibria to Eq*. (4): $(v_{1},v_{2},\theta)=\{(0,0,0),(1,1,1), \mathbf{z}_{1}\equiv (1/\rho , 0, 1/\rho), \mathbf{z}_{2} \equiv(0,1/(1-\rho),1/(1-\rho))\}$. *The first two are always unstable*. *Let* $a=|\mathbf{x}_{2}|^{2}$, $b=\mathbf {x}_{1}\cdot\mathbf{x}_{2}$, $c=\rho/(1-\rho)$, *and* $\tau=\tau _{\theta}/\tau_{w}$. *Then*

- $\mathbf{z}_{1}$ *is linearly asymptotically stable if and only if*
  $$c\bigl(a-b^{2}\bigr) (1-ac)\tau^{2} -\bigl(1+2ac-a^{2}c^{2}-2b^{2}c \bigr)\tau+(1+ac) > 0. $$
- $\mathbf{z}_{2}$ *is linearly asymptotically stable if and only if*
  $$c\bigl(a-b^{2}\bigr) (a-c)\tau^{2}+\bigl(2c \bigl(b^{2}-a\bigr)+c^{2}-a^{2}\bigr)\tau+ a+c > 0. $$
- *If* $a=1$ (*that is*, *the stimuli have equal amplitude*), *then* $\mathbf{z}_{1,2}$ *are linearly asymptotically stable if and only if*
  $$c(1-c) \bigl(1-b^{2}\bigr)\tau^{2} +\bigl(2c \bigl(b^{2}-1\bigr)+c^{2}-1\bigr)\tau+ 1+c > 0. $$
- *In the simplest case where* $a=c=1$, *then both selective equilibria are stable if and only if*
  $$\tau< \frac{1}{1-b^{2}}. $$

### Bifurcation Analysis

**Fig. 4:**
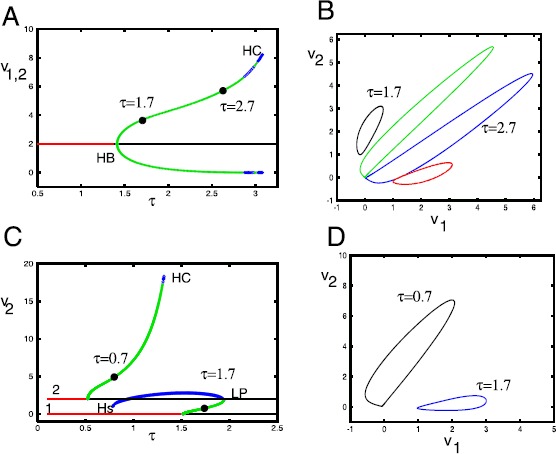
Behavior of Eq. (4) as $\tau=\tau _{\theta}/\tau_{w}$ changes. $\mathbf{x}^{(1)}=(1,0)$, $\mathbf{x}^{(2)}=A(\cos(1),\sin(1))$, $\rho=1/2$. (**A**) $A=1$, so that both fixed points have the same stability properties. *Curves* show maximum and minimum value of $v_{1}$ or $v_{2}$. *Red line* shows stable equilibrium, *black*, unstable equilibrium, *green circle* show stable limit cycles and *blue* unstable. Two points are marked by *black filled circles* and the Hopf bifurcation is depicted as *HB*. Apparent homoclinic is labeled *HC*. (**B**) Symmetric pairs of limit cycles for two different values of *τ* on the curves in (**A**) projected on the $(v_{1},v_{2})$ plane. (**C**) $A=1.5$ so that the stability of the two equilibria is different. The maximum value of $V_{2}$ is shown as *τ* varies. *Upper curves* (2) bifurcate from $(v_{1},v_{2},\theta )=(0,2,2)$ and *lower curves* (1) from $(2,0,2)$. Colors as in panel A. *LP* denotes a limit point and *Hs* denotes a Hopf bifurcation for the symmetric equilibrium $(1,1,1)$. (**D**) Orbits taken from the two bifurcation curves in (**C**) projected onto the $(v_{1},v_{2})$ plane

The previous section shows that as the ratio *τ* increases, the two selective equilibria lose stability via a Hopf bifurcation. We now use numerical methods to study the behavior as *τ* increases. As the stability theorem shows, if the amplitude of the two stimuli are the same, then the stability is exactly the same for both, no matter what the other parameters. We will fix $\rho=0.5$, and $\mathbf {x}^{(1)}=(1,0)$, $\mathbf{x}^{(2)}=A(\cos(1),\sin(1))$ and let *τ* vary. In Fig. 4A, we show the case $A=1$ so that both stimuli have the same magnitudes. As *τ* increases, each of the selective equilibria loses stability at the same value of *τ*, here given by $\tau_{c}=1/(1-\cos(1)^{2})=1.412$ (cf. Eq. (8)). At this point a stable limit cycle bifurcates and exists up until $\tau \equiv\tau_{HC}\approx3.2$ where the orbit appears to be homoclinic to the nonlinear saddle at the origin. (Note that near the homoclinic, there are some numerical issues with the stability; we believe that the branch is stable all the way up to the homoclinic.) We remark that the dynamics for *τ* slightly larger than $\tau_{HC}$ is difficult to analyze; while the origin is unstable, it has stable directions and it appears that all initial data eventually converge to it. For *τ* large enough, we have found that solutions blow up in finite time.

If the amplitude of $\mathbf{x}^{(2)}$ is different from that of $\mathbf{x}^{(1)}$, then the theorem shows that the two selective equilibria have different stability properties. Figure 4C shows the bifurcation diagram for $A=1.5$. When we follow the stability of $\mathbf{z}_{1}=(2,0,2)$ (shown as the lower curve labeled 1), there is a Hopf bifurcation at $\tau\approx1.52$ and a stable branch of periodic orbits bifurcates from it that persists up until $\tau\approx1.94$ where it bends around (LP), becomes unstable, and terminates on the symmetric unstable equilibrium, $(v_{1},v_{2},\theta)=(1,1,1)$ at a Hopf bifurcation ($\tau\approx0.79$) for this equilibrium, labeled Hs. Figure 4D shows the small amplitude periodic orbit at $\tau=1.7$ projected in the $(v_{1},v_{2})$ plane where it is centered around $(v_{1},v_{2})=(2,0)$. The upper curve in panel C (labeled 2) shows the stability of $\mathbf{z}_{2}=(0,2,2)$ as *τ* varies. Here, there is a Hopf bifurcation at $\tau\approx0,5$ and a stable branch of periodic orbits bifurcates from the equilibrium. The branch terminates at a homoclinic orbit at $\tau\approx1.35$. Figure 4D shows an orbit for $\tau=0.7$ that surrounds $(v_{1},v_{2})=(2,0)$.

In sum, in this section we have analyzed a very simple BCM model where there are two stimuli, two weights, and one neuron. We have shown that if the time-scale factor ($\tau_{\theta}$) of the homeostatic threshold, *θ* is too slow relative to the time-scale factor of the weights, then, the selective equilibria lose stability via a Hopf bifurcation and limit cycles emerge. For very large ratios, $\tau=\tau_{\theta}/\tau _{w}$, solutions become unbounded and intermediate values of *τ*, the origin becomes an attractor even though it is unstable. In the next section, we consider the case when there are more stimuli than there are weights and, in the subsequent section, we consider small coupled networks.

## Results II: One Neuron, *n* Weights, *m* Stimuli

We next consider the general scenario where a single neuron receives an *n*-dimensional input selected from *m* different possibilities with probability $p_{k}$, $k=1,\ldots,m$. We will label the stimuli $x_{kj}$ with *j* running from $1,\ldots,n$, and *k* as above. The weights are $w_{1},\ldots,w_{n}$ and the response of a neuron to stimulus *k* is

10 $$ v_{k} = \sum_{j=1}^{n} w_{j} x_{kj}. $$

If the weights and the threshold change slowly compared to the change in the stimulus presentation, then the differential equations for the BCM rule can be averaged over the inputs:

$$\begin{aligned} \tau_{w} w_{j}' = \sum _{k=1}^{m} p_{k} x_{kj}v_{k}(v_{k}- \theta), \qquad \tau_{\theta}\theta' = \sum_{k=1}^{m} p_{k} v_{k}^{2} - \theta, \end{aligned}$$

where $v_{k}$ is given in Eq. (10). We note that, classically, what is of interest is not the evolution of the weights, but rather the evolution of the responses. Using Eq. (10), we see that

11 $$\begin{aligned} \tau_{w} v_{k}' =& \tau_{w}\sum_{j=1}^{n} x_{kj} w_{j}' = \sum_{j=1}^{n} \sum _{l=1}^{m} x_{kj}x_{lj}p_{l} v_{l}(v_{l}-\theta) \\ =& \sum_{l=1}^{m} p_{l} \vec{x}_{k}\cdot\vec{x}_{l} v_{l}(v_{l}- \theta), \end{aligned}$$

where $\vec{x}_{k}$ is the vector whose entries are $(x_{k1},\ldots ,x_{kn})$. It is very clear that using this formulation, the equations are very simple. Let *X* denote the matrix whose entries are $x_{kj}$; it is an $m\times n$ matrix. If $n=m$, then *X* is square, and if it is invertible, then the two formulations with respect to the weights and the responses are equivalent. That is, $\vec{v}(t)=X \vec{w}(t)$. However, if $n\ne m$, then there will be some degeneracy with respect to the two formulations. Most typically, the dimension of the stimulus space will be larger than the dimension of the weight space ($m>n$) and in this case there will be degeneracy with respect to the responses. As should be clear from the two formulations, the equations are much simpler in the response space, so that this is the preferred set of ODEs and thus there will be redundancy in the equations. That is, there will be $m-n$ linearly independent vectors, $\vec{q}_{i}$ such that $\vec{q}_{i}^{T} X=0$. This implies that here will be $m-n$ constants of motion in the response space:

12 $$ \vec{q}_{i} \cdot\vec{v}(t) = C_{i}. $$

Thus, in the case when $m>n$, we still need only study the $n+1$-dimensional dynamical system consisting of *n* choices of the $v_{k}$ along with the $m-n$ linear constraints (12).

### Example: $n=2,m=3$

As an example of the kinds of dynamics that is possible, we will consider $m=3$ and $n=2$ where the three stimuli are $(1,0),(\cos \alpha ,\sin\alpha)$, and $(\cos\beta,\sin\beta)$ and these are distributed with equal probability. In this case, the equations for $v_{k},\theta$ are

13 $$\begin{aligned} &\tau_{w} v_{k}' = \frac{1}{3}\sum_{l=1}^{3} c_{kl}v_{l}(v_{l}-\theta), \\ &\tau_{\theta}\theta' = -\theta+ \frac{1}{3}\sum _{l=1}^{3} v_{l}^{2}, \end{aligned}$$

with $c_{ll}=1$, $c_{lk}=c_{kl}$, $c_{12}=\cos\alpha$, $c_{13}=\cos \beta $, and $c_{23}=\cos(\alpha-\beta)$. Since there are two weights and three stimuli, we can reduce the dimension by 1 with the constraint:

$$e_{1}v_{1}+e_{2}v_{2}+e_{3}v_{3}=C, $$

where $e_{1}=\cos\alpha\cos(\alpha-\beta)$, $e_{2}=-\sin\alpha\sin \beta$ and $e_{3}=\sin(\alpha)^{2}$. As long as one of these is non-zero (which will happen if the vectors are not all collinear), we can solve for one of the $v_{k}$ and reduce the dimension by 1. In the example that we analyze here, we fix $\alpha=0.92$ and $\beta=2.5$ and eliminate $v_{3}$. This leaves two parameters, $\tau\equiv\tau_{\theta}/\tau_{w}$ and *C*, the constant of integration. Equilibria are independent of *τ* but the existence of limit cycles and other complex dynamics obviously depends on *τ*.

**Fig. 5:**
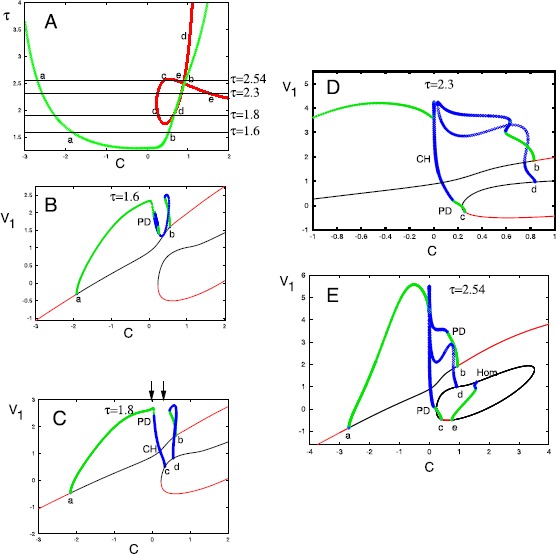
Bifurcation diagrams for Eq. (13) as the constant *C* varies for different values of the ratio $\tau=\tau_{\theta}/\tau_{w}$. (**A**) Summary of the possible Hopf bifurcations on the principal branch (*green*) and on the isola (*red*). Labels correspond to different branches of Hopf bifurcations on the panels that follow. (**B**–**E**) the maximum value of $v_{1}(t)$ as a function of *C* for different values of the ratio *τ*. *Thin black lines* are unstable equilibria, *red* are stable equilibria, *green* and *blue circles* are stable and unstable limit cycles. *PD* is for period-doubling bifurcation; *CH* for chaos, *HOM* for homoclinic. *Arrows* in C correspond to chaotic behavior shown in Fig. 6

**Fig. 6:**
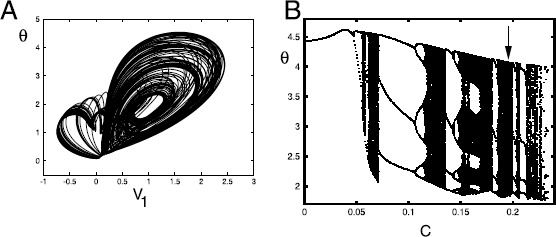
(**A**) Chaos in Eq. (13) for $\tau=1.8$ and $C=0.18$ projected in the $v_{1}-\theta$ plane. (**B**) Orbit diagram obtained by taking a Poincaré section at $v_{2}=2$ and plotting successive values of *θ* as *C* varies. An *arrow* denotes $C=0.18$; cf. panel A

Figure 5 shows the dynamics as *C* is varied for different values of the ratio *τ*. Panel E shows the full range of equilibria as the constant, *C* varies. For large negative values of *C*, there is a unique equilibrium point and for $C\in(0.235,3.65)$ there are two additional equilibria formed by an isola (isolated circle) of equilibria. The stability of all of these equilibria depends on the values of *τ* and *C*. The change in stability occurs when there is a Hopf bifurcation. Panel A shows a summary in two parameters of the curves of Hopf bifurcation points. The green curve corresponds to the stability of the upper branch of equilibria in panels B–E. For $\tau<1.293$, there are no Hopf bifurcations on either branch and there appear to be no periodic orbits. For all $\tau>1.293$, the upper branch has two Hopf bifurcations (labeled a, b) so that we can expect the possibility of periodic behavior. The curve of the Hopf bifurcations is more complicated for the isola. We first note that the upper part of the isola always has one real positive eigenvalue, so that it is unstable for all *τ*. The lower part of the isola has a negative real eigenvalue and its stability depends on *τ*. Returning to the Hopf bifurcations on the isola of equilibria (shown in red in panel A), we see that there can be 1, 2 or 3 Hopf bifurcations as *C* changes. We label these c, d, e. Since there are generally two Hopf bifurcations on the main branch of equilibria, there can be up to five Hopf bifurcations for a given value of *τ* as *C* increases. We start with $\tau=1.6$ (panel B). For this value of *τ*, we see it is below the minimum for which there are Hopf bifurcations on the isola, so all the bifurcations appear on the main branch. Both bifurcations are supercritical and lead to small amplitude stable oscillations that grow in amplitude. The branches of periodic orbits arising from the two Hopf bifurcations are joined and thus represent a single continuous branch. However, the branch starting at a loses stability via a period-doubling bifurcation (PD in panel B) at $C\approx0.177$. There does not appear to be any chaotic behavior that we have been able to find. For $\tau =1.8$, shown in panel C, we see that the branch of periodic orbits that bifurcated from the main branch (at points a, b), has split into two separate branches that terminate on Hopf bifurcations of the upper branch of the isola (points c, d). The left branch that joins a and c also undergoes a period-doubling bifurcation (PD) and for a limited range of *C*, there appears to be chaos in the dynamics; specifically around $C=0.18$. Two arrows delimit the range of parameters that are shown in Fig. 6. For $\tau=2.3,2.54$, there are 5 Hopf bifurations and as with $\tau=1.8$ the periodic orbits arising from point a join with those on point c and those arising from b join with the branch arising from d. The branch of stable periodic orbits arising from the Hopf bifurcation at e is lost at a homoclinic labeled Hom in panel E. There is a small regime of chaotic behavior for $\tau=2.3$ shown in panel D, but we find no chaos when $\tau=2.54$, For larger values of *τ*, there are three Hopf bifurcations (a, b, d). The bifurcations c,e merge and disappear so that all the equilibria on the isola are unstable. The branch of periodic orbits arising from d, becomes disconnected from the branch arising from b while the branch of orbits arising ftom b joins the branch arising from a. Other than the unique stable equilibrium when *C* is large or small, there is only a principal branch of stable periodic orbits between the Hopf bifurcations a and b. There are other complex structures, but none of them are stable.

Figure 6 shows some probable chaos for $\tau=1.8$ and $C\in[0,0.25]$. Panel A shows a trajectory projected in the $v_{1}-\theta $ plane for $C=0.18$. Panel B shows the evolution of the attracting dynamics as *C* varies. We take a Poincaré section at $v_{2}=2$ and plot the successive values of *θ* after removing transients and letting *C* vary between 0 and 0.25. As *C* increases, there is a periodic orbit that undergoes multiple period-doubling bifurcations before becoming chaotic. There are several regions showing period three orbits ($C\approx0.1$, $C\approx0.175$, $C\approx0.21$) as well as many regions with complex behavior. The chaos and periodic dynamics terminates near $C=0.237$, which is the value of *C* at which the lower stable branch of equilibria in the isola begins. Chaos and similar complex dynamics occurs for other values of *τ*.

In this section, we have shown that the degeneracy that occurs when there are more stimulus patterns than weights can be resolved by finding some simple constants of motion. The resulting reduced system will always be three-dimensional. In the simplest case of three patterns and two weights, we have found rich dynamics when $\tau=\tau _{\theta}/\tau_{w}$ is larger than 1.

## Results III: Small Coupled Network

To implement a network of coupled BCM neurons receiving stimulus patterns from a common set, it is important to incorporate a mechanism for competitive selectivity within the network. A mechanism of this sort, found in visual processes [23] (and also in tactile [24], auditory [25], and olfactory processing [26]) is called lateral inhibition, during which an excited neuron reduces the activity of its neighbors by disabling the spreading of action potentials to neighboring neurons in the lateral direction. This creates a contrast in stimulation that allows increased sensory perception.

Consider a network of *N* mutually inhibiting neurons. At any time, let $\{ v_{i}\}_{i=1}^{N}$ be the net activities of the neurons. Let $\{ s_{i}\} _{i=1}^{N}$ be the partial activities induced by a stimulus **x** i.e. $s_{i}= \mathbf{w}_{i} \cdot\mathbf{x}$ where $\mathbf{w}_{i}$ is the synaptic weight vector for neuron *i*. At any given time, the activity, $v_{i}$ of the linear neuron *i* (where $i \in\{ 1, 2, \ldots,N\}$) follows the differential equation:

$$\frac{dv_{i}}{dt} = -v_{i} + I_{i}, $$

where $I_{i}$ is the external input to the neuron [27]. Since neuron *i* is inhibited by its neighbors

$$I_{i} = s_{i} - \gamma\sum_{j\neq i} v_{j}, $$

where *γ* is the inhibition parameter, measuring the amount of inhibition that *i* gets. Therefore

14 $$ \frac{dv_{i}}{dt} = -v_{i}+s_{i} - \gamma \sum_{j\neq i} v_{j}. $$

At a steady state, this equation becomes

15 $$ s_{i} = v_{i} + \gamma\sum _{j\neq i} v_{j}. $$

Thus, the overall activity of the network can be expressed as

$$\mathbf{v} = G^{-1} \mathbf{s}, $$

where

$$G= \begin{bmatrix} 1 & \gamma& \gamma& \ldots& \gamma\\ \gamma& 1 & \gamma& \ldots& \gamma\\ \gamma& \gamma& 1 & \ldots& \gamma\\ \vdots& \vdots& \vdots& \ddots& \vdots\\ \gamma& \gamma& \gamma& \ldots& 1 \end{bmatrix}.$$

Now let $V=\sum_{j=1}^{N} v_{j}$. Then we can write Eq. (15) as

$$v_{i}= s_{i} -\gamma V + \gamma v_{i} $$

or

16 $$ v_{i} = \frac{s_{i} -\gamma V}{1-\gamma} ; $$

thus

$$\begin{aligned} V =& \sum_{j=1}^{N} \frac{s_{j}-\gamma V}{1-\gamma} = \frac{1}{1-\gamma} \Biggl( \sum_{j=1}^{N} s_{j} -\gamma\sum_{j=1}^{N} V \Biggr) \\ =& \frac{1}{1-\gamma} \Biggl( \sum_{j=1}^{N} s_{j} -\gamma NV\Biggr), \end{aligned}$$

implying

$$\bigl(1+\gamma(N-1)\bigr)V= \sum_{j=1}^{N} s_{j} $$

or

$$V= \frac{\sum_{j=1}^{N} s_{j} }{1+ \gamma(N-1)} . $$

Substituting *V* into Eq. (16) we get

$$v_{i} = \frac{1}{1-\gamma}s_{i} - \frac{\gamma}{(1-\gamma)(1+\gamma(N-1))} \sum _{j=1}^{N} s_{j}. $$

The left-hand side of this equation is undefined when $\gamma=1$ or $\gamma=-\frac{1}{N-1}$. Thus *G* is invertible when $0<\gamma<1$.

Linearizing around the steady state solution of Eq. (14), we obtain the Jacobian

$$M= \begin{bmatrix} -1 & -\gamma& -\gamma& \ldots& -\gamma\\ -\gamma& -1 & -\gamma& \ldots& -\gamma\\ -\gamma& -\gamma& -1 & \ldots& -\gamma\\ \vdots& \vdots& \vdots& \ddots& \vdots\\ -\gamma& -\gamma& -\gamma& \ldots& -1 \end{bmatrix}. $$

Notice that $(1, 1, \ldots,1)^{T}$ is an eigenvector of *M* with corresponding eigenvalue $-(1+ N \gamma)$. This eigenvalue is negative when $\gamma>-1/N$. Also notice that *M* can be written as  where  is the $N-by-N$ matrix of all 1’s and  is the $N-by-N$ identity matrix. Note that , since  and .  is in the eigenspace of *M* because if  then  Thus *u* is an eigenvector of *M* corresponding to the eigenvalue $\gamma-1$. This eigenvalue is negative when $0<\gamma<1$. Thus whenever *G* is invertible, the system is also stable.

Now consider two neurons **a** and **b** who mutually inhibit each other and, at any instant, receive the same stimulus pattern **x**, with synaptic weight vectors $\mathbf{w}_{\mathbf{a}}$ (for neuron **a**) and $\mathbf{w}_{\mathbf{b}}$ (for neuron **b**). Let their responses to **x** be $s_{a}$ and $s_{b}$, and their net responses (after accounting for inhibition) be $v_{a}$ and $v_{b}$. Finally, let the dynamic threshold to $v_{a}$ and $v_{b}$ be $\theta_{a}$ and $\theta_{b}$, respectively. The BCM learning rule of these two neurons is given by

17 $$ \begin{aligned} \tau_{w}\dot{\mathbf{w}}_{a} &= \mathbf{x}v_{a}(v_{a}-\theta _{a}), \\ \tau_{\theta}\dot{\theta}_{a} &= {v_{a}}^{2} -\theta_{a}, \\ \tau_{w}\dot{\mathbf{w}}_{b} &= \mathbf{x}v_{b}(v_{b}- \theta _{b}), \\ \tau_{\theta}\dot{\theta}_{b} &= {v_{b}}^{2} -\theta_{b}, \end{aligned} $$

where $s_{a} = \mathbf{w}_{\mathbf{a}}\cdot\mathbf{x}$ and $s_{b} = \mathbf{w}_{\mathbf{b}}\cdot\mathbf{x}$ and thus

18 $$ \begin{bmatrix} v_{a} \\ v_{b} \end{bmatrix} = \begin{bmatrix} 1 & \gamma\\ \gamma& 1 \end{bmatrix} ^{-1} \begin{bmatrix} s_{a} \\ s_{b} \end{bmatrix} $$

or

19 $$ \begin{aligned} v_{a}&= \frac{1}{1-\gamma^{2}} s_{a} - \frac{\gamma}{1-\gamma^{2}}s_{b}, \\ v_{b}&= \frac{-\gamma}{1-\gamma^{2}} s_{a} + \frac{1}{1-\gamma^{2}}s_{b}. \end{aligned} $$

### Mean Field Model

Consider the general two-dimensional stimulus pattern $\mathbf{x}= (x_{1}, x_{2})$. Let the two neurons, **a** and **b**, receive this stimulus with the synaptic weight vectors $\mathbf{w}_{\mathbf{a}}=(w_{a1}, w_{a2})$ and $\mathbf{w}_{\mathbf{b}}=(w_{b1}, w_{b2})$. If $g = 1/(1-\gamma^{2})$ and $h = \gamma /(1-\gamma^{2})$, then according to Eq. (19)

20 $$ \begin{aligned} v_{a}&= c_{a1}x_{1} + c_{a2}x_{2}, \\ v_{b}&=c_{b1}x_{1} + c_{b2}x_{2}, \end{aligned} $$

where $c_{a1} = g w_{a1} -hw_{b1}$, $c_{a2}= gw_{a2} -h w_{b2}$, $c_{b1}= g w_{b1} - h w_{a1}$ and $c_{b2}= g w_{b2} - h w_{a2}$.

The rate of change of $v_{a}$ is given by

21 $$\begin{aligned} \dot{v}_{a} =& \dot{c}_{a1}x_{1} + \dot{c}_{a2}x_{2} \\ =& g x_{1} \dot{w}_{a1} - h x_{1} \dot{w}_{b1} + g x_{2} \dot{w}_{a2} -h x_{2} \dot{w}_{b2} \\ =& g( x_{1} \dot{w}_{a1} + x_{2} \dot{w}_{a2}) - h (x_{1}\dot{w}_{b1} + x_{2}\dot{w}_{b2}). \end{aligned}$$

A similar expression exists for $v_{b}$. Assume that **x** is from the set $\{ \mathbf{x}^{(1)}=(x_{11}, x_{12}), \mathbf{x}^{(2)} =(x_{21}, x_{22})\}$ such that $Pr[\mathbf{x}(t)=\mathbf{x}^{(1)} ]= \rho$ and $Pr[\mathbf{x}(t)=\mathbf{x}^{(2)} ]=1-\rho$. Then in terms of the responses, the mean field version of the BCM rule for two mutually inhibiting neurons **a** and **b** is derived as follows:

22 $$ \begin{aligned} \tau_{\theta}\dot{\theta}_{a} ={}& \rho v_{a1}^{2} + (1-\rho) v_{a2}^{2} - \theta_{a}, \\ \tau_{w} \dot{v}_{a1} ={}& g \bigl[ \rho \mathbf{x}^{(1)} \cdot\mathbf{x}^{(1)} v_{a1}( v_{a1}-\theta_{a}) + ( 1-\rho) \mathbf{x}^{(1)} \cdot\mathbf{x}^{(2)} v_{a2}( v_{a2}- \theta_{a}) \bigr] \\ &{}- h \bigl[ \rho\mathbf{x}^{(1)} \cdot\mathbf{x}^{(1)} v_{b1}( v_{b1}-\theta _{b}) + ( 1-\rho) \mathbf{x}^{(1)} \cdot\mathbf{x}^{(2)} v_{b2}( v_{b2}-\theta _{b}) \bigr], \\ \tau_{w} \dot{v}_{a2} ={}& g \bigl[ \rho \mathbf{x}^{(2)} \cdot\mathbf{x}^{(1)} v_{a1}(v_{a1}- \theta_{a}) + ( 1-\rho) \mathbf{x}^{(2)} \cdot \mathbf{x}^{(2)} v_{a2}(v_{a2}-\theta_{a}) \bigr] \\ &{}- h \bigl[ \rho\mathbf{x}^{(2)} \cdot\mathbf{x}^{(1)} v_{b1}( v_{b1}-\theta _{b}) + ( 1-\rho) \mathbf{x}^{(2)} \cdot\mathbf{x}^{(2)} v_{b2}(v_{b2}- \theta _{b}) \bigr], \\ \tau_{\theta}\dot{\theta}_{b} ={}& \rho v_{b1}^{2} + (1-\rho) v_{b2}^{2} - \theta_{b}, \\ \tau_{w} \dot{v}_{b1} ={}& g \bigl[ \rho \mathbf{x}^{(1)} \cdot\mathbf{x}^{(1)} v_{b1}( v_{b1}-\theta_{b}) + ( 1-\rho) \mathbf{x}^{(1)} \cdot\mathbf{x}^{(2)} v_{b2}( v_{b2}- \theta_{b}) \bigr] \\ &{}- h \bigl[ \rho\mathbf{x}^{(1)} \cdot\mathbf{x}^{(1)} v_{a1}( v_{a1}-\theta _{a}) + ( 1-\rho) \mathbf{x}^{(1)} \cdot\mathbf{x}^{(2)} v_{a2}(v_{a2}- \theta _{a}) \bigr], \\ \tau_{w} \dot{v}_{b2} ={}& g \bigl[ \rho \mathbf{x}^{(2)} \cdot\mathbf{x}^{(1)} v_{b1}( v_{b1}-\theta_{b}) + ( 1-\rho) \mathbf{x}^{(2)} \cdot\mathbf{x}^{(2)} v_{b2}( v_{b2}- \theta_{b}) \bigr] \\ &{}- h \bigl[ \rho\mathbf{x}^{(2)} \cdot\mathbf{x}^{(1)} v_{a1}(v_{a1}-\theta _{a}) + ( 1-\rho) \mathbf{x}^{(2)} \cdot\mathbf{x}^{(2)} v_{a2}( v_{a2}-\theta _{a}) \bigr]. \end{aligned} $$

Observing that each of *ρ*, $\mathbf{x}^{(1)}$, and $\mathbf {x}^{(2)}$ is non-zero, and setting the right-hand side of Eq. (22) to zero yields

$$\begin{aligned} \rho v_{a1}^{2} + (1-\rho) v_{a2}^{2} - \theta_{a} &=0, \\ \rho v_{b1}^{2} + (1-\rho) v_{b2}^{2} - \theta_{b} &= 0, \\ v_{a1}(v_{a1} - \theta_{a}) &= 0, \\ v_{a2}(v_{a2} - \theta_{a}) &= 0, \\ v_{b1}(v_{b1} - \theta_{b}) &= 0, \\ v_{b2}(v_{b2} - \theta_{b}) &= 0. \end{aligned}$$

Solving this system of equations gives the set of fixed points $(v_{a1}, v_{a2}, \theta_{a}, v_{b1}, v_{b2}, \theta_{b}) = \{(0, 0, 0, 0, 0, 0), (\frac{1}{\rho}, 0, \frac{1}{\rho} , \frac{1}{\rho}, 0, \frac {1}{\rho} ), ( 0, \frac{1}{1-\rho}, \frac{1}{1-\rho}, 0, \frac {1}{1-\rho}, \frac{1}{1-\rho} ), (\frac{1}{\rho}, 0, \frac{1}{\rho} , 0, \frac{1}{1-\rho}, \frac {1}{1-\rho} ), (0, \frac{1}{1-\rho}, \frac{1}{1-\rho} ,\frac{1}{\rho}, 0, \frac{1}{\rho} ), (1, 1, 1, 1, 1,1), (1,1,1,\frac{1}{\rho},0,\frac{1}{\rho}), \ldots\}$. The … in these fixed points correspond to the symmetric variants of the last equilibrium, for example swapping the $(1,1,1)$ and the $(\frac{1}{\rho},0,\frac{1}{\rho})$ or swapping the latter triplet for $(0,\frac{1}{1-\rho},\frac{1}{1-\rho})$.

Castellani et al. [9] and Intrator and Cooper [7] give a detailed analysis on the stability of most of these fixed points in the limit of $\tau_{\theta}\to0$. They showed that $(0,0,0, 0,0,0)$ and $(1,1,1, 1,1,1)$ are unstable and the fully selective fixed points are stable. This leaves the fixed points of the form $(1,1,1,\frac {1}{\rho},0,\frac{1}{\rho})$. We address these below for our particular choice of stimuli.

We will explore the dynamics of Eq. (22) as $\tau =\tau _{\theta}/\tau_{w}$ changes in a very simple scenario in which, $\rho=0.5$, and $\mathbf{x}^{(1)}=(1,0)$ and $\mathbf{x}^{(2)}=(\cos\alpha,\sin \alpha)$. In this case, there are only two equilibria that need to be studied: the *symmetric* case $(\theta_{a},v_{a1},v_{a2},\theta _{b},v_{b1},v_{b2})= (2,2,0,2,2,0)$ and the *antisymmetric* case, $(2,2,0,2,0,2)$. The other selective equilibria are symmetric to these two. In the symmetric case, neurons a and b are both selective to stimulus 1 and in the antisymmetric case, neuron a selects stimulus 1 and neuron b selects stimulus 2. Fixing *α*, the angle between the stimuli leaves two free parameters, *τ* and *γ*, the inhibitory coupling.

### Stability of the Selective Equilibria

We now consider the stability of these equilibria in the simplified case of the previous paragraph ($\rho=0.5$, $\mathbf{x}^{(1)},\mathbf {x}^{(2)}$ are unit vectors with $\mathbf{x}^{(1)}\cdot\mathbf {x}^{(2)}=\beta =\cos(\alpha)$ where *α* is the angle between them). We exploit the symmetry of the resulting equations to factor the characteristic polynomial into the product of two cubic polynomials and then use the Routh–Hurwitz criteria. We have made use of the symbolic capabilities of Maple. Again, let $\beta=\cos(\alpha)$, $g=1/(1-\gamma^{2})$ and $h=\gamma/(1-\gamma^{2})$. The linearization of the symmetric equilibrium $(v_{a1},v_{a2},\theta_{a},v_{b1},v_{b2},\theta_{b})=(2,0,2,2,0,2)$ is

$$M_{s} = \begin{pmatrix} g & -\beta g & -g & -h & \beta h & h \\ \beta g & -g & -\beta g & -\beta h & h & \beta h \\ 2/\tau&0 & -1/\tau& 0 & 0 & 0 \\ -h & \beta h & h & g & -\beta g & -g \\ -\beta h & h & \beta h & \beta g & -g & -\beta g \\ 0 & 0 & 0 & 2/\tau& 0 & -1/\tau \end{pmatrix}. $$

This matrix is clearly block symmetric with $3\times3$ blocks $G,H$. The the stability is thus found by studying $M_{1}=G+H$ and $M_{2}=G-H$. Let $a_{1}=g-h$ and $a_{2}=g+h$. Then the blocks have the form

$$M_{j} = \begin{pmatrix} a_{j} & -\beta a_{j} & -a_{j} \\ \beta a_{j} & -a_{j} & -\beta a_{j} \\ 2/\tau&0& -1/\tau \end{pmatrix}. $$

The characteristic polynomial of $M_{j}$ is

$$\lambda^{3} + \frac{1}{\tau} \lambda^{2} + \frac{1}{\tau }\bigl(a_{j}\bigl(2+a_{j}\tau \bigl( \beta^{2}-1\bigr)\bigr)\bigr)\lambda+\frac{a_{j}^{2}(1-\beta^{2})}{\tau}. $$

Clearly the $\lambda^{2}$ coefficient and the constant coefficient are positive. The *λ*- coefficient could become negative if $\tau >2/(a_{j}(1-\beta^{2}))\equiv\tau_{j1}^{s}$. The Routh–Hurwitz criterion also requires

$$2a_{j}\bigl(1+a_{j}\tau\bigl(\beta^{2}-1\bigr) \bigr)>0. $$

This quantity becomes negative for $\tau> 1/(a_{j}(1-\beta^{2}))\equiv \tau _{jH}^{s}$. Clearly $\tau_{jH}^{s}<\tau_{j1}^{s}$, so as *τ* increases there will be a Hopf bifurcation. Since $0< a_{1}< a_{2}$, we see that the symmetric equilibrium will be stable if and only if

$$\tau< \tau_{2H}^{s} = \frac{1-\gamma}{1-\cos^{2}\alpha}, $$

where we have used the definitions of $g,h,\beta$. The critical value of *τ* is a linear function of *γ* and this critical value can be arbitrarily small as $\gamma\to1$. We also remark that the critical instability is due to $a_{2}$, which is associated with $G-H$. Thus, we expect a *symmetry-breaking Hopf* bifurcation to out-of-phase oscillations. We will numerically confirm this result in the subsequent numerical analysis.

We can do a similar calculation for the antisymmetric equilibrium. Here, we just state the final result; the approach and calculations are similar. The characteristic polynomial factors. Each factor has the form

$$P_{\pm}(\lambda)=\lambda^{2} + \frac{1}{\tau} \lambda^{2} +\biggl[2\frac{g\pm \beta h}{\tau}-\bigl(1-\beta^{2} \bigr) \bigl(g^{2}-h^{2}\bigr)\biggr]\lambda+ \frac{(1-\beta ^{2})(g^{2}-h^{2})}{\tau}. $$

The constant coefficient and the quadratic coefficient are always positive. The linear coefficient is positive as long as

$$\tau< \tau_{2\pm}=\frac{g\pm\beta h}{(1-\beta^{2})(g^{2}-h^{2})}\equiv 2 \tau _{\pm}^{a}. $$

The additional Routh–Hurwitz condition is positive if and only if

$$\tau< \tau_{\pm}^{A}, $$

which is clearly less than $\tau_{2\pm}$. Applying the definitions of $g,h,\beta$, yields

$$\tau_{\pm}^{a} = \frac{1\pm\gamma\cos(\alpha)}{1-\cos^{2}(\alpha)}. $$

Clearly $\tau_{-}^{A}<\tau_{+}^{a}$, so that the antisymmetric solution is stable as long as $\tau< \tau_{-}^{a}\equiv\tau_{H}^{a}$.

We summarize the stability results in the following theorem.

#### Theorem 5.1

*Assume that the two stimuli are unit vectors with an angle* $\alpha\ne 0,\pi$ *and are presented with equal probability*. *Then*

1. *The symmetric equilibrium*, $(v_{a1},v_{a2},\theta _{a},v_{b1},v_{b2},\theta_{b})=(2,0,2,2,0,2)$ *is linearly asymptotically stable if and only if*
  $$\tau< \tau_{H}^{s} = \frac{1-\gamma}{1-\cos^{2}(\alpha)}. $$
  *Furthermore the unstable direction is antisymmentric*.
2. *The antisymmetric equilibrium* $(v_{a1},v_{a2},\theta _{a},v_{b1},v_{b2},\theta_{b})=(2,0,2,0,2,2)$ *is linearly asymptotically stable if and only if*
  $$\tau< \tau_{H}^{a} = \frac{1-\gamma\cos(\alpha)}{1-\cos^{2}(\alpha)}. $$
  *Furthermore the unstable direction is symmetric*.

We remark that, for acute angles where $\cos(\alpha)>0$, the symmetric equilibrium loses stability at lower values of *τ* than does the antisymmetric equilibrium and for obtuse angles ($\cos\alpha<0$) it is vice versa.

*Partially selective equilibria.* Using the same notation as for the selective equilibria, we consider the partially selective fixed points for $\rho=1/2$ (e.g. $(1,1,1,2,0,2)$ etc.). An elementary evaluation of the constant coefficient of the characteristic polynomial yields a value:

$$a_{0}=-\bigl(g^{2}-h^{2}\bigr)^{2} \bigl(\beta^{2}-1\bigr)^{2}/\bigl(4\tau^{2}\bigr), $$

which is clearly negative. Since the product of the eigenvalues is $a_{0}$ this implies that the eigenvalues have mixed signs and these equilibria are saddle points.

### Numerical Results

In this section, we study the numerical behavior of Eq. (17) for $\rho=0.5$, $\mathbf{x}^{1,2}$ unit vectors with angle $\alpha=0.7709$ as *τ* and *γ* vary. We will generally set $\gamma=0.25$. The choice for *α* is somewhat arbitrary but was found to yield rich dynamics.

**Fig. 7:**
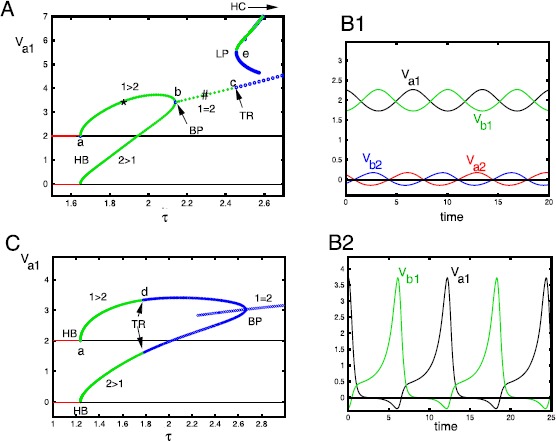
Bifurcation of the symmetric state, $(v_{a1},v_{a2},\theta _{a},v_{b1},v_{b2},\theta_{b})=(2,0,2,2,0,2)$, as *τ* increases for two values of *γ*. (**A**) $\gamma=0.2$. *Red lines* represent the stable equilibrium, *black lines* are unstable. *Green* (*blue*) *thick lines* are stable (unstable) periodic orbits. *HB*: Hopf bifurcation; *BP*: Branch point; *TR*: Torus bifurcation; *LP*: Limit point; *HC*: Homoclinic orbit. (**B1**, **2**) Representative periodic solutions at the points labeled with a *star* and a *sharp sign*. (**C**) Same as (**A**), but $\gamma=0.4$. Note that the upper periodic orbit is not shown in this diagram

We first study the behavior of the symmetric equilibrium $(v_{a1},v_{a2},\theta_{a},v_{b1}, v_{b2},\theta_{b})=(2,0,2,2,0,2)$ as *τ* increases. In Fig. 7A, we set $\gamma=0.2$. For *τ* small enough, the selective symmetric equilibrium is stable, with increasing *τ* loses stability and we have a Hopf bifurcation (HB). A branch of periodic solutions emerges where $v_{*1}>v_{*2}$ for each neuron, $*=\{a,b\}$. At a critical value of *τ* there is a branch point (or pitchfork) bifurcation (BP) where this selective periodic solution intersects a non-selective periodic solution. The selective periodic solutions have either $v_{*1}>v_{*2}$ ($1>2$, top branch) or ($2>1$, lower branch). The non-selective branch (with a # on it) loses stability at a torus bifurcation (TR). Beyond the torus, there are, at first, stable non-selective quasi-periodic solutions, and then some possible chaos. We will look at chaotic solutions when we describe the antisymmetric behavior. Figures 7B1, 2 show the *V*’s for the selective and non-selective stable oscillations at values of *τ* denoted by the ⋆, and the ♯ ($\tau=1.55,\tau=2.29$, respectively). In Fig. 7C, we set $\gamma=0.4$ and see a behavior similar to panel A, but the selective branches lose stability at a torus bifurcation at values of *τ* less than the branch point and this gives rise to attracting quasi-periodic selective behavior, and then, for *τ* a bit larger, selective chaos. For all *γ*, when *τ* is larger than about 3, the solutions produce a “spike” and then return to 0. We know that the origin is unstable, but there are some stable directions and all solutions appear^1^ to approach this stable direction when *τ* is large enough.

**Fig. 8:**
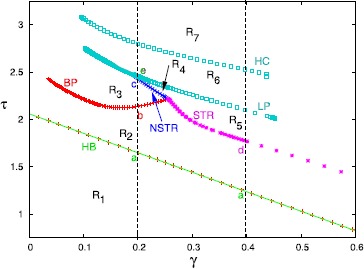
Two-parameter diagram corresponding to Fig. 7 that divides $(\tau,\gamma)$ into different regions. Labels as in Fig. 7, with NSTR corresponding to a non-selective torus bifurcation and STR, the selective one. Small letters, *a*, *b*, *c*, *d*, *e* correspond to the letters in Fig. 7. See text for details

**Fig. 9:**
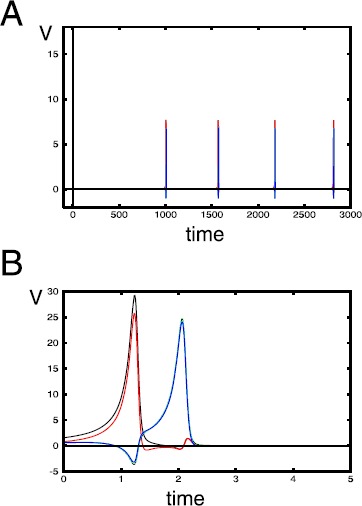
$(v_{a1},v_{a2},v_{b1},v_{b2})$ for *τ* in regions 6 and 7; $\alpha=0.7709$, $\gamma=0.2$ (**A**) $\tau=2.65$ (**B**) $\tau=3$

Figure 8 summarizes the behavior of the symmetric branch of solutions as *τ* and *γ* vary. Specific bifurcation points from Fig. 7 are labeled a, b, c, d in this figure. There are seven regions in the diagram. In R1, the equilibrium is stable; this region is delineated by the Hopf bifurcation (HB) line that we computed in Theorem 5.1, $\tau_{HB}^{S}=(1-\gamma)/(1-\cos^{2}\alpha)$. Region R2 corresponds to a non-symmetric periodic orbit such as shown in Fig. 7B1. If *γ* is small (roughly, less than 0.26), then, as *τ* increases, there is a reverse pitchfork bifurcation (BP) to a non-selective periodic orbit (R3) such as shown in Fig. 7B2. This orbit loses stability at the non-selective torus bifurcation (NSTR) as we enter R4. In R4, there is quasi-periodic and chaotic behavior, but the dynamics lies on the four-dimensional space $V_{a1}=V_{a2},V_{b1}=V_{b2}$. Passing from R2 to R5 also appears to lead to quasi-periodic and chaotic behaviors. Region R6, delineated below by the curve of limit points (LP) above, by an apparent homoclinic orbit (HC) consists of large amplitude stable periodic orbits where $V_{a1}=V_{a2}$ and $V_{b1}=V_{b2}$. This branch of solutions (seen in the one-parameter diagram, Fig. 7A at the top right) does not connect to the other branches until *γ* is close to zero (not shown). As *τ* increases, the period of these orbits appears to go to infinity and they spend more and more time near the origin. We find that in R7, the origin is a global attractor, even though it is unstable. Figures 9A,B show simulations when *τ* is in R6 (panel A) and in R7 (panel B). Initial conditions were chosen with no special symmetry. In Fig. 9A, we see a brief transient, followed by a gap and then, eventually long period activity. In Fig. 9B, we only show the first five time units, but after $t=20{,}000$, we still saw no return to activity.

**Fig. 10:**
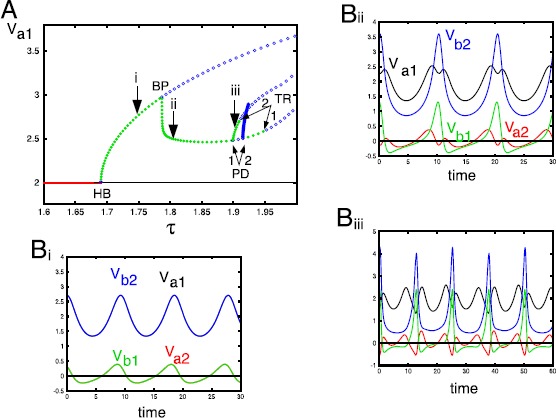
(**A**) Bifurcation diagram for the antisymmetric state $(v_{a1},v_{a2},\theta_{a},v_{b1},v_{b2},\theta_{b})=(2,0,2,0,2,2)$ as *τ* increases for $\gamma=0.25$. Abbreviations as in Fig. 7 and *PD*: Period doubling. **B**(**i**–**iii**) Roman numerals correspond to the solutions shown in (**A**). In **B**(**i**), $v_{a1}=v_{b2}$ and $v_{a2}=v_{b1}$

We next turn to the behavior of the antisymmetric equilibrium, $(v_{a1},v_{a2},\theta_{a},v_{b1}, v_{b2},\theta_{b}) =(2,0,2,0,2,2)$ as *τ* increases. Figure 10A shows the fate of this branch of solutions as *τ* increases for $\gamma=0.25$ and $\alpha=0.7709$. As described in Theorem 5.1, there is a Hopf bifurcation at $\tau=(1-\gamma\cos\alpha)/(1-\cos^{2}\alpha)$ and this gives rise to a branch of periodic orbits (labeled i). Figure 10B(i) shows a time series of the $v_{a1,b2,a2,b1}$ which maintains the symmetry of $v_{a1}=v_{b1}$ and $v_{a2}=v_{b1}$. At a critical value of *τ* there is symmetry-breaking bifurcation and a new branch of solutions emerges where all the *v*’s are different. This is shown in Fig. 10B(ii). Further increases in *τ* lead to a pair of period-doubling bifurcations, PD1, PD2. The branch emerging from PD1 leads to a stable periodic branch, an example of which is in Fig. 10B(iii). The second branch, PD2, leads to an unstable branch of solutions and re-stabilizes the branch labeled ii. This branch and the branch labeled iii then lose stability at torus bifurcations, labeled TR1 and TR2, respectively. Below, we will explore what happens after the bifurcation at TR2. Once *τ* gets large enough, the dynamics appears to become symmetric with $v_{a1}=v_{a2}$ and $v_{b1}=v_{b2}$ where it is as in Fig. 9.

**Fig. 11:**
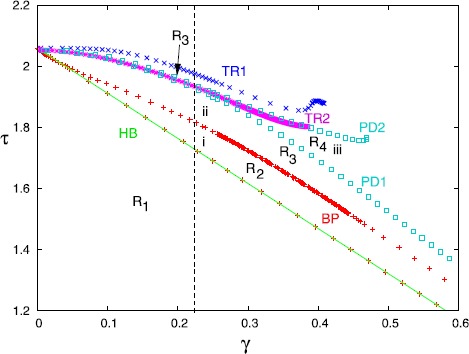
Two-parameter diagram corresponding to Fig. 10. Labels (*i*–*iii*) correspond to the three points shown in Fig. 10. Torus and period-doubling bifurcations are shown. See text for full details

Figure 11 shows the behavior of the antisymmetric branch as *τ* and *γ* change. For a fixed value of *γ*, as *τ* increases, the selective state ($R_{1}$) loses stability at the Hopf bifurcation (HB) at $\tau=(1-\gamma\cos\alpha)/(1-\cos^{2}\alpha)$ as shown in Theorem 5.1. The branch of periodic orbits such as seen in Fig. 10B(ii) is found in $R_{2}$ and loses stability at a pitchfork bifurcation (BP). The HB and BP curves appear sequentially for all $\gamma<1$ in contrast to Fig. 8. In the region $R_{3}$, solutions have lost the symmetry of $R_{2}$ and resemble the solutions shown in Fig. 10B(ii). Further increases in *τ* lead to a periodic doubling and solutions in the region $R_{4}$ look like Fig. 10B(iii). Region $R_{4}$ is bounded by PD1 and the torus bifurcation TR2 for $\gamma<0.375$. For $\gamma>0.375$, instead of a torus bifurcation, there is a period-doubling cascade to chaos (not shown). Beyond the torus bifurcation, there seems to be quasi-periodic motion that persists until PD2 where the branch labeled ii stabilizes again to form a new region $R_{3}$. This branch loses stability at a torus bifurcation TR1. For *τ* beyond TR1, there seems to be chaos, quasi-periodic behavior, and complex periodic orbits. Eventually, for *τ* large enough, the dynamics of Fig. 9 is all that remains.

## Discussion

We have explored the BCM rule as a dynamical system. Although the literature does not suggest a homeostatic time-scale range that ensures stability of a biological system, we have shown that the selective fixed points of the BCM rule are generally stable when the homeostatic time scale is faster than synaptic modification time scale, and that some complex dynamics emerges as the homeostatic time scale varies. The nature of this complex dynamics also depends on the angular and amplitudinal relationships between stimuli in the stimulus set. In our analysis, the neuron is presented with stimuli that switch rapidly, so it was possible to reduce the learning rule to a simple averaged set of differential equations. We studied the dynamics and bifurcation structures of these averaged equations when the homeostatic time scale is close to the synaptic modification time scale, and found that instabilities arise, leading to oscillations and in some cases chaos and other complex dynamics. Similar results would hold if the quadratic term $v^{2}$ in the second line of Eq. (2) were replaced with $v^{p}, p>2$, since the original formulation by Bienenstock et al. [5] suggests that the fixed point structures are preserved for any positive value of *p*. Since the onset of the bifurcations (such as the Hopf bifurcation) depends mainly on the symmetry of these fixed points, we expect that the main results will be the same and only the particular values of parameters would change. While this paper has focused on how small changes in the time scale of a homeostatic threshold can lead to complex dynamics, there are many other kinds of homeostases [28] which present many time scales and similar opportunities for analysis.

The model neuron we used in this paper has been assumed to have linear response properties, which may be seen as oversimplified, and hence a potential problem in translating our conclusions to actual biological systems. It is well known that plasticity goes beyond synapses, and it is sometimes even a neuron-wide phenomenon [29], and that there is no unique route to regulating the sliding threshold of the BCM rule [10, 30]. Thus in addition to synaptic activities, intrinsic neuronal properties may also play a role in the evolution of responses and linearity may not be able to capture this scenario. The introduction of a nonlinear transfer function to the BCM learning rule has been addressed by Intrator and Cooper [7]. In their formulation, the learning rule is derived as a gradient descent rule on an objective function that is cubic in the nonlinear response. Our decision to use linear units is motivated by the accessibility to formal analysis. Biologically, linearity can be justified if we assume that the underlying biochemical mechanisms are governed by membrane voltage rather than firing rate; see, for example Clopath and Gerstner [31].

The theoretical contributions of this paper are based on an analysis that we did using a mean field model of the BCM learning rule. Similar mean field models have been made, but in terms of synaptic weights; see, for example Yger and Gilson [32]. With this approach to the mean field, it is difficult to arrive at the fact that the fixed points—not their stability—of the learning rule depend only on the probabilities with which each stimulus is presented. In this paper, we have given a derivation of the mean field model of the BCM learning rule as a rate of change of the activity response *v*, with time. The derived model considers the amplitudes of the stimuli presented, the pairwise angular relationships between the stimuli, and the probabilities with which the stimuli are presented. The appeal of this derivation is that it easily highlights the fact that the fixed points depend on these probabilities. Additionally, the derivation is important because the dynamics of the BCM learning rule is driven by the activity response (not the synaptic weights), and many analyses in the literature rely on this fact; see, for example Castellani et al. [9]. Our analyses considered three cases: one neuron with two weights and two stimuli, one neuron with two weights and three stimuli, and lastly a weakly interacting small network of neurons.

In exploring the dynamics of a single neuron, we used Fig. 3 to show the dependence of critical value $\tau_{c}$ of $\tau =\tau_{\theta}/\tau_{w}$—which leads to a Hopf bifurcation—on the angle, *α* between the stimuli, the amplitudinal factor, *A* between the stimuli, and the probability distribution, *ρ* of the stimuli. The role of *τ* as a bifurcation parameter has been seen in several recent works including Zenke et al. [33], Toyoizumi et al. [34], and Yger and Gilson [32]. A possible future work, which is beyond the scope of this paper, is to investigate the dependence of the selectivity of the neuron on *τ*. For a single neuron presented with a set of stimuli *S*, Bienenstock et al. [5] defined the selectivity of the neuron as a function of the area under the tuning curve of the neuronal responses to *S*. This definition, however, assumes that the learning rule converges to a stable steady state. To analyze the selectivity of a neuron as *τ* varies, one would need a measure of selectivity that addresses an oscillatory steady state. Thus, it might be more appropriate to talk about relative selectivity (RS) in this case. If the neuron receives stimulus inputs from the set $S=\{\mathbf{x}^{(1)}= (x_{11}, x_{12} ), \mathbf{x}^{(2)}= (x_{21}, x_{22}) \}$ with synaptic weights $\mathbf{w}=(w_{1},w_{2})$, then at any point in time, $v_{1}(t) = w_{1}(t)x_{11} + w_{2}(t)x_{12}$ and $v_{2}(t) = w_{1}(t)x_{21}+ w_{2}(t)x_{22}$. If for given *τ*, we let $t_{o}$ be the point in time at which the dynamics of the neuron achieves a stable steady state or an oscillatory steady state, and $d(\tau) = \min_{t \geq t_{o}} |v_{1}(t)-v_{2}(t)| $, then we can define RS as follows:

$$RS(\tau) =\frac{d(\tau)}{\underset{ \tau_{1} \in(0, \infty)}{\max} d(\tau_{1})}. $$

Note that $0\leq RS \leq1$, since it is defined as a fraction of the maximum selectivity. For this reason it tends to have the same maximum value and shape for all values of $\alpha\in(-\pi/4, \pi/4)$. Preliminary analysis of this formulation allows us to conjecture that RS stays pretty much at its maximum for $\tau\in(0, \tau_{c})$ decays to 0 as *τ* increases on $(\tau_{c}, \infty)$.

In our analysis of a small network (see Sect. 5) we have made the simplifying assumption that the lateral inhibitory weight is constant in time. The incorporation of an inhibitory plasticity rule (as in Moldakarimov et al. [35]) would necessitate a third time-scale parameter, and possibly a fourth if the inhibitory rule were to include a dynamic modification threshold. This is beyond the scope of the paper and reserved for future work. Another related possible future direction is to perform an analysis of a large network of BCM neurons, by observing what happens to the network dynamics at different time-scale parametric regimes. A good starting point is to explore the dynamics for a fully connected network with equal inhibition, that is, each neuron is coupled with every other neuron in the network and inhibits each of them equally. The next step would be to let the amount of inhibition vary according to how far away the inhibiting neuron is. It may also be interesting to examine how the architecture of the network is affected. We know, for instance, that spike-time dependent plasticity (STDP) has the ability to yield a feedforward network out of a fully connected network. The analysis that Kozloski and Cecchi [36] used to demonstrate this finding centers around the synaptic weights. Thus it will be useful to pay closer attention to the synaptic weights in future work. Moreover, the oscillatory and chaotic properties we observed in the small coupled network will also be observed had our mean field been derived in terms of the weight and the analyses been done with the synaptic weights.

The debate about synaptic homeostatic time scales in neurobiology remains vibrant. A review of the literature seems to reveal a varied, and somewhat paradoxical set of findings among experimentalists and theoreticians. While homeostasis of synapses found in experiments is slow [12, 37], homeostasis of synapses in most theoretical models needs to be rapid and sometimes even instantaneous to achieve stability [33, 38, 39]. There are, however, ongoing efforts to shed more lights on the debate. It has been suggested that both fast and slow homeostatic mechanisms exist. Zenke and Gerstner [39] suggest that learning and memory use an interplay of both forms of homeostasis; while fast homeostatic control mechanisms help maintain the stability of synaptic plasticity, slower ones are important for fine-tuning neural circuits. In addition to the present work contributing to the debate by demonstrating the relevance of fast homeostasis to synaptic stability, it also furthers the discussion as regards the link between STDP and the BCM rule: Zenke et al. [33] found that homeostasis needs to have a faster rate of change for spike-timing dependent plasticity to achieve stability. Furthermore it is well known that, under certain conditions, the BCM learning rule follows directly from STDP [13, 14].

## Footnotes

1 We have no proof of this, but we have observed it in every choice of parameters.
